# High-resolution fluorodeoxyglucose positron emission tomography and magnetic resonance imaging findings of a pituitary microtumor in a dog

**DOI:** 10.1186/s13620-015-0050-5

**Published:** 2015-09-23

**Authors:** Young-Don Son, Da-Jung Kim, Ji-Houn Kang, Dong-Woo Chang, Young-Bae Jin, Dong-In Jung, Chulhyun Lee, Mhan-Pyo Yang, Sang-Rae Lee, Byeong-Teck Kang

**Affiliations:** Neuroscience Research Institute, Gachon University, Incheon, South Korea; Department of Biomedical Engineering, College of Health Science, Gachon University, Incheon, South Korea; Laboratory of Veterinary Dermatology and Neurology, College of Veterinary Medicine, Chungbuk National University, Cheongju, Chungbuk South Korea; Laboratory of Veterinary Internal Medicine, College of Veterinary Medicine, Chungbuk National University, Cheongju, Chungbuk South Korea; Laboratory of Veterinary Radiology, College of Veterinary Medicine, Chungbuk National University, Cheongju, Chungbuk South Korea; The National Primate Research Center, Korea Research Institute of Bioscience and Biotechnology (KRIBB), Ochang, Chungbuk Republic of Korea; Institute of Animal Medicine, Gyeongsang National University, Jinju, South Korea; Center of Magnetic Resonance Research, Korea Basic Science Institute, Ochang, Chungbuk Republic of Korea

**Keywords:** Dog, Fluorodeoxyglucose positron emission tomography, Magnetic resonance imaging, Pituitary microtumor

## Abstract

A 16-year-old, castrated, male English cocker spaniel dog was presented due to generalized alopecia. Routine clinical pathology, endocrine and abdominal ultrasonography results were consistent with a diagnosis of pituitary-dependent hyperadrenocorticism. The adenohypophyseal lesion was clearly visualized on both 3 T and 7 T magnetic resonance imaging (MRI) of the pituitary gland. Although biochemical and MRI findings were consistent with a functional pituitary microtumor, a pituitary lesion was not detected using ^18^F-fluorodeoxyglucose positron emission tomography (FDG-PET). This report firstly describes the application of high-resolution FDG-PET to a spontaneous pituitary microtumor in a dog.

## Background

Hyperadrenocorticism is a common endocrine disorder in dogs, whose primary causes are pituitary and adrenocortical tumors [1, 2]. However, as many as 80–85 % of the patients with hyperadrenocorticism are believed to have chronic excessive secretion of adrenocorticotropic hormone (ACTH) from a neoplasm within the pituitary gland [3].

Classically, pituitary tumors have been divided into microtumors (less than 10 mm in greatest diameter) and macrotumors (over 10 mm in greatest diameter) [4]. More than half of dogs with pituitary-dependent hyperadrenocorticism (PDH) have microtumors [5]. The neuroradiologic evaluation of pituitary tumors is currently based on computed tomography (CT) or magnetic resonance imaging (MRI), with or without contrast medium enhancement [6]. Pituitary macrotumors are well identified by CT or MRI [7], but microtumors often cannot be visualized in dogs because of small variation in pituitary gland size and shape [8].

In human medicine, MRI with high-field strength (1.5 to 3 T) is considered to be the most sensitive imaging modality for the detection of pituitary microtumors [9]. However, up to 40 % of confirmed PDH cases have negative results in MRI examinations [10, 11]. To overcome this low detection rate, positron emission tomography (PET) with ^18^F-fluorodeoxyglucose (FDG) has been used in the diagnosis of microtumors. Previous studies demonstrated that FDG-PET has the potential to provide valuable clinical information in the assessment of pituitary microtumors, especially in difficult cases of biochemically confirmed PDH when other MRI studies are negative or questionable [10, 12, 13].

Because of the potential value of FDG-PET for detecting microtumors and limited PET data in dogs, this imaging technique was applied in the present case of a pituitary microtumor. This case report firstly demonstrates the high-resolution PET characteristics of a pituitary microtumor in a dog.

## Case presentation

### Diagnosis of PDH

A 16-year-old, castrated male English Cocker spaniel dog was presented with a 4 year history of dermatological problems such as alopecia and pruritus. Polyuria and polydipsia were not noted by the client. Physical examination revealed a distended abdomen, generalized alopecia, and seborrheic dermatitis. Increased alkaline phosphatase activity (ALP: 660 IU/L, reference range: 29–97 IU/L) was observed on the serum chemistry panel. The levels of total cholesterol (221 mg/dL, reference range: 135–270 mg/dL) and triglycerides (76 mg/dL, reference range: 21–116 mg/dL) were within the reference range. Hepatomegaly and bilateral enlarged adrenal glands of relatively equal size (left: 6.2 mm, right: 5.9 mm in thickness of the cranial poles) were identified on abdominal radiographs and ultrasonography, respectively. Because these findings suggested hyperadrenocorticism, an ACTH stimulation test and a high-dose dexamethasone suppression test (HDDST) were performed. The serum samples were obtained for the ACTH stimulation test before and 1 h after intravenous administration of 0.25 mg synthetic ACTH (Synacthen, Novartis Pharma, Basel, Switzerland); pre-ACTH cortisol concentration was 226.2 nmol/L (reference range: 14–166 nmol/L) and post-ACTH concentration was 717.3 nmol/L (reference range: 166–469 nmol/L). Serum cortisol concentration was suppressed after administration of dexamethasone (0.1 mg/kg intravenously [IV]; Je-Il Pharm, Daegu, South Korea): pre-HDDST cortisol concentration was 69.5 nmol/L and 4-h and 8-h post-HDDST cortisol concentrations were 40 nmol/L and <27.6 nmol/L, respectively. Based on these results, a diagnosis of PDH was made.

### FDG-PET and 7 T MRI fusion imaging

To evaluate the pituitary gland region, intracranial imaging was performed using a high-resolution research tomography (HRRT)-PET and 7 T MRI fusion imaging system, with the owner’s permission. Written client consent was obtained prior to examination, and this procedure was performed with the approval of our institutional review board committee. The dog was fasted for 12 h, and then injected with FDG (0.4 mCi/kg IV); the dog was kept caged for 1 h to minimize movement and ensure a stable FDG uptake. The FDG-PET scan was conducted for 30 min on the HRRT device (resolution, 2.5 mm full width at half maximum resolution in three-dimensional [3D] acquisition mode; ECAT HRRT; Siemens, Knoxville, TN, USA) under general anesthesia maintained by tiletamine/zolazepam (8 mg/kg IV; Zoletil; Virbac, Carros, France) following medetomidine (20 μg/kg intramuscularly [IM]; Domitor; Pfizer, Seoul, South Korea) premedication. Immediately after the PET scan, the shuttle system transported the dog to 7 T-MRI scanner (Magnetom 7 T; Siemens, Berlin, Germany), and then pre- and post-contrast T1-weighted 3D magnetization-prepared rapid gradient echo images (TR: 3000 ms, TE: 2.98 ms, TI: 1100 ms, flip angle: 10°, matrix: 192 × 256 × 256, field of view [FOV]: 96 × 128 mm^2^, slab thickness: 128 mm) and transverse T2-weighted turbo spin echo images (TR: 3000 ms, TE: 72 ms, flip angle: 60°, matrix: 384 × 384, FOV: 100 mm, slice thickness: 1.5 mm, interslice gap: 0 mm) of the brain were obtained. To minimize personnel radiation exposure, the dog was remotely monitored in the approved holding facility during the recovery from anesthesia.

**Fig. 1:**
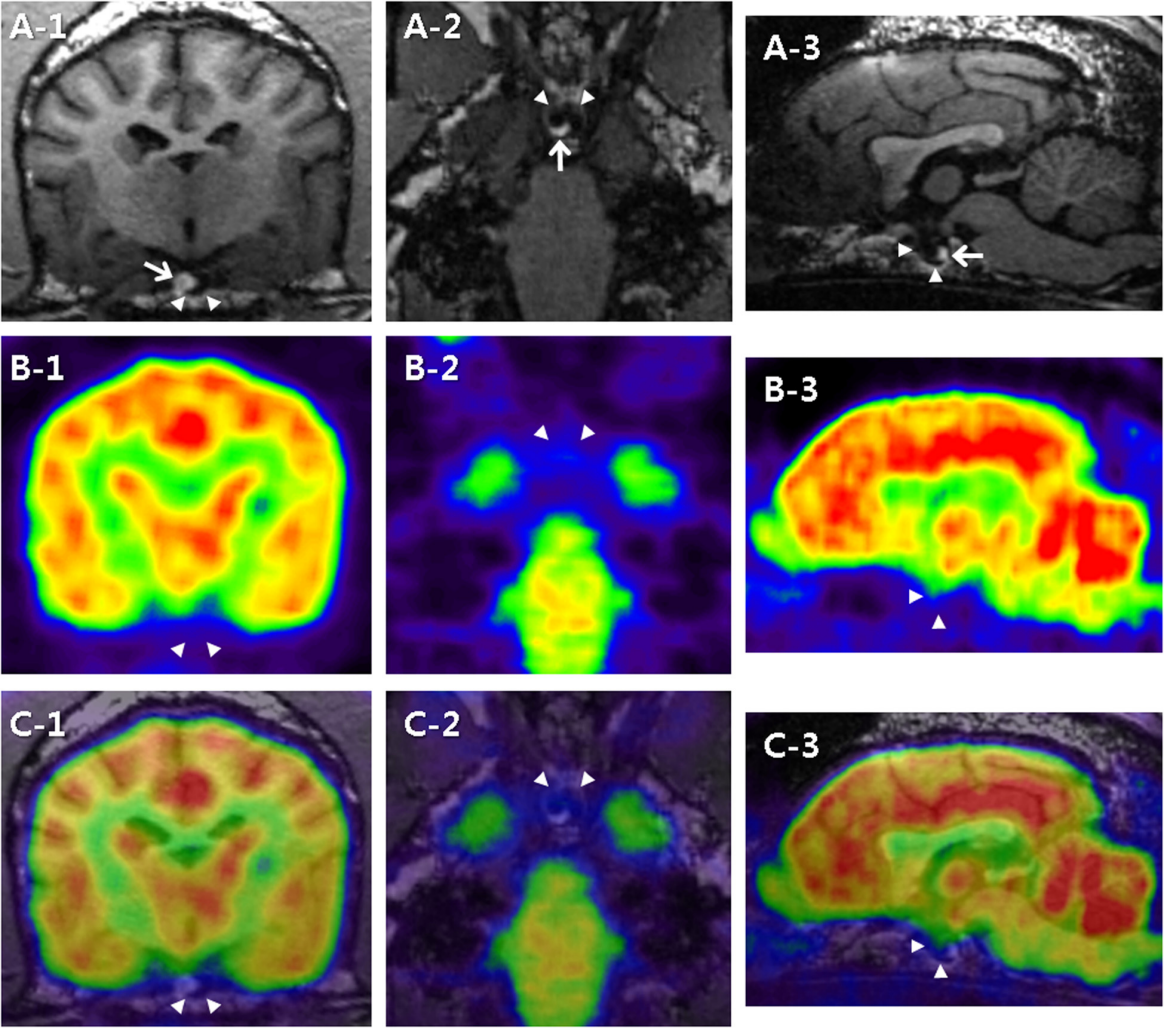
FDG-PET and 7 T MRI characteristics of a canine pituitary microtumor. Transverse (1), dorsal (2), and sagittal (3) images at the level of the pituitary gland are pictured from left to right. **a** On pre-contrast T1-WI, the hyperintense neurohypophysis (*arrow*) was displaced in the left-dorsal direction by the arcuate adenohypophysis with hypointensity (*arrowheads*). **b** On PET images, high FDG uptake is represented by *reddish* to *yellowish color*, while low uptake is represented by *bluish* to *greenish color*. Glucose metabolism of the pituitary gland (*arrowheads*) was relatively lower than that of the cerebral cortex and the white matter. **c** The anatomical location of the pituitary gland (arrowheads) was clearly identified by combining 7 T MRI images with PET images

**Fig. 2:**
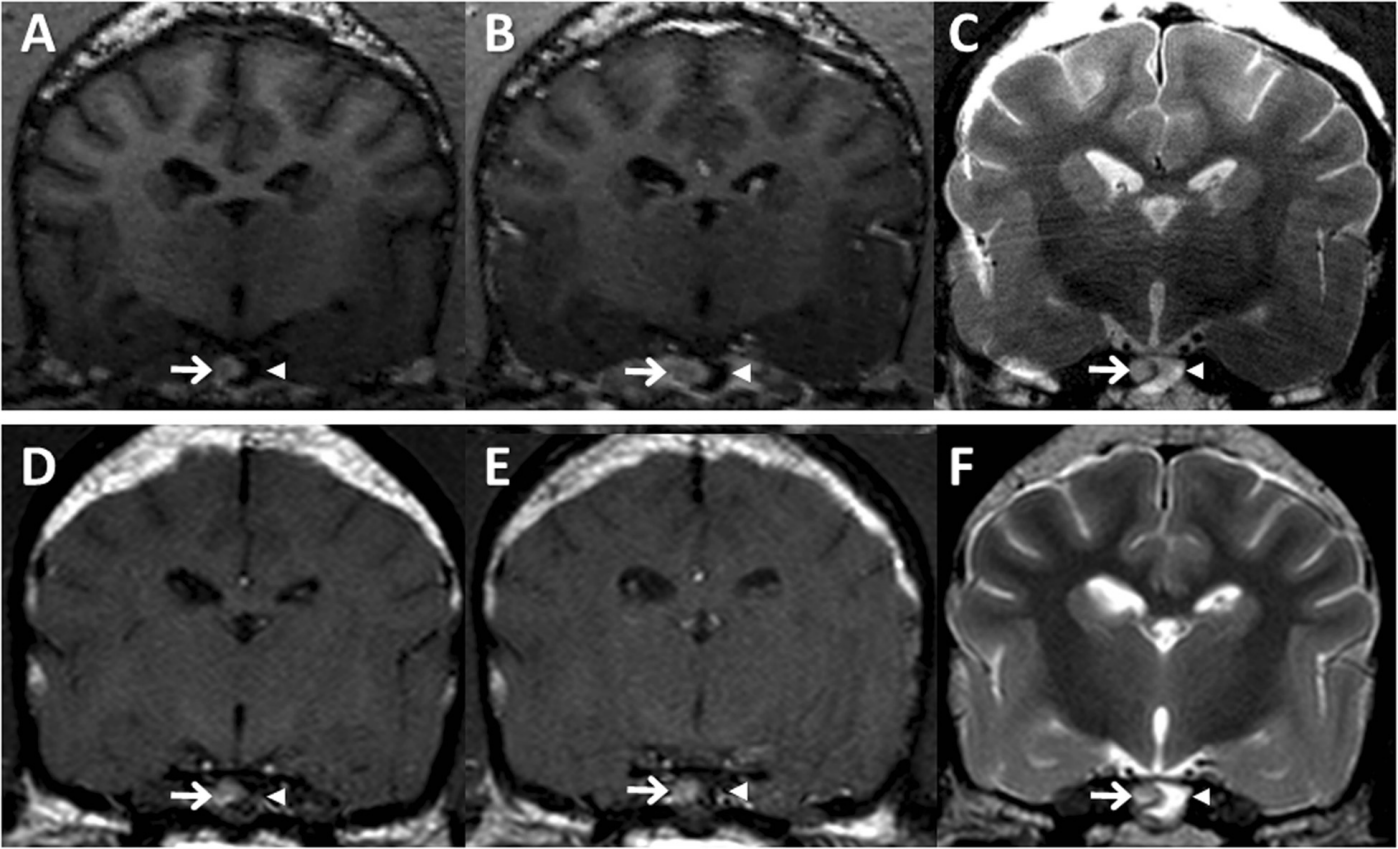
High-resolution MRI characteristics of a canine pituitary microtumor. Following initial 7 T MRI scan (**a**–**c**), a pituitary lesion was re-evaluated by 3 T MRI 6 months later (**d**–**f**). **a** Pre-contrast T1-WI showed the hypointense adenohypophyseal lesion (*arrowhead*), indicating a pituitary microtumor, and the hyperintense neurohypophysis (*arrow*). **b** Uniform contrast enhancement was observed on the neurohypophysis (*arrow*), while the adenohypophyseal lesion had no enhancement (arrowhead) on post-contrast T1-WI. **c** T2-WI revealed hyperintensity in the adenohypophyseal lesion (arrowhead) and isointensity in the neurohypophysis (*arrow*). At 6 month follow-up, MRI findings of the adenohypophyseal lesion (*arrowhead*) and the neurohypophysis (*arrow*) were not changed on pre- (**d**) and post- (**e**) contrast T1-WI and T2-WI (**f**), except for a slight increase in the size of the pituitary gland

On MRI, an arcuate lesion was noted in the right-ventral part of the pituitary gland. It had hypointensity on pre-contrast T1-weighted images (WI) (Figs. 1a and 2a) and hyperintensity on T2-WI (Fig. 2c). This adenohypophyseal lesion displaced the neurohypophysis in the left-dorsal direction. Pre-contrast T1- and T2-WI of the neurohypophysis showed hyperintensity (Figs. 1a and 2a) and isointensity (Fig. 2c), respectively. Following administration of gadolinium-diethylenetriamine pentaacetic acid (0.1 mmol/kg IV; Omniscan; Nycomed, Princeton, NJ, USA), signal intensity was increased uniformly in the neurohypophysis, while the adenohypophyseal lesion had no enhancement (Fig. 2b). The height of the pituitary gland was 4.16 mm and pituitary height/brain area (P/B) ratio was 0.3. These MRI findings were consistent with pituitary microtumor. The FDG uptake of the pituitary gland was not elevated on HRRT-PET scan (Fig. 1b and c). The standardized uptake value (SUV) was lower in the pituitary gland (SUV: 1.23) than in the gray matter (SUV: 3.95). The anatomical location of the pituitary gland was more precisely identified by the PET-MRI fusion images (Fig. 1c).

### Follow-up MR scan

The dog was treated with trilostane (3 mg/kg orally [PO], twice daily [BID]; Vetoryl; Dechra, Shrewsbury, UK), and then post-ACTH cortisol concentration had been maintained between 2 and 5 μg/dL. Six months after initial MRI, a pituitary lesion was monitored by 3 T MRI scan (Achieva 3.0 T multi TX; Philips Healthcare, Best, NL) with following sequences: pre- and post-contrast T1-weighted spin echo imaging (TR: 500 ms, TE: 12 ms, flip angle: 90°, matrix: 256 × 256, FOV: 150 mm, slice thickness: 3 mm, interslice gap: 0 mm) and T2-weighted turbo spin echo imaging (TR: 4000 ms, TE: 80 ms, flip angle: 90°, matrix: 304 × 294, FOV: 130 mm, slice thickness: 3 mm, interslice gap: 0 mm). Although the height (4.67 mm) and P/B ratio (0.31) of the pituitary gland were slightly increased, other MRI findings were not different from the first MRI scan (Fig. 2d–f). Presently, the dog is managed well without dermatological or neurological abnormalities.

## Conclusions

Typical MRI characteristics of pituitary microtumors include the displaced neurohypophysis and the adenohypophyseal lesion with hypointensity and hyperintensity on T1- and T2-WI, respectively [5, 8, 14]. Although the normal pituitary gland has a rapid contrast enhancement due to the lack of a blood–brain barrier, signal intensity of a pituitary tumor is usually low after the injection of the contrast media [14, 15]. In the present case, these features of a pituitary microtumor were noted on MRI scans, which were performed twice with a 6 month interval. Pituitary enlargement was not identified on either of the two MRI scans, using previously reported reference intervals for height and P/B ratio (height: 3–7.5 mm; P/B ratio: ≤0.31) [16, 17]. Canine pituitary tumors are classified as adenomas, invasive adenomas, and adenocarcinomas [7, 18]. A previous MRI study demonstrated that tumor size is the only variable that determines the type of pituitary tumors in dogs [7]. In that study, adenomas were the smallest tumors; lack of contrast enhancement was observed in 10 % of adenomas. In another study, 1 year follow-up MRI of the pituitary gland showed no apparent change of tumor size in half of dogs with a visible tumor on the initial MRI scan [19]. Even though the present case had the imaging features of adenomas, the characterization of tumor type was impossible because of no histologic data.

High-field MRI is the imaging technique of choice in humans with pituitary tumors because of its superior contrast resolution. In human patients with PDH, the sensitivity of 3 T MRI in detecting pituitary microtumors is relatively higher than that of 1.5 T MRI [20]. In addition, 3 T and 7 T MRI provide good spatial and contrast resolution for the identification of clinically relevant brain anatomy in dogs [21, 22]. In the present case, the two high-field MRI scanners clearly showed a pituitary lesion because the image quality for T1- and T2-WI was comparable at 3 T and 7 T. Further investigation in a large number of dogs is needed to evaluate the usefulness of 3 T and 7 T-MRI owing to the lack of high-field MR data in the diagnosis of pituitary tumors.

Although CT and MRI have been commonly used in the diagnosis of PDH, detection of pituitary lesions still remains a problem. Pituitary tumors are detectable by CT or MRI in less than half of humans and dogs with PDH [8, 10, 11, 15, 23]. Previously, increased FDG uptake values were noted in human cases of pituitary microtumors with no abnormal CT or MRI findings [10, 12, 13]. The ability of FDG-PET to detect pituitary macrotumors has been well recognized in human medicine, whereas the FDG-PET appearance of microtumors is controversial because of frequently observed negative findings [10, 13, 24]. In the present case, a pituitary lesion was not visualized on FDG-PET, even though MRI and biochemical characteristics were typical for a functional ACTH-secreting microtumor. Macrotumors are usually functional, locally invasive, and can grow rapidly, while microtumors are typically slow-growing and noninvasive [7]. A previous study showed that FDG uptake values are higher in pituitary macrotumors than in microtumors [13]. Additionally, it was reported that the FDG uptake values of nonfunctional pituitary tumors are higher than those of functional pituitary tumors [13, 25, 26]. Therefore, FDG uptake, shown by PET imaging, may be more directly related to tumor growth rate than hormonal secretion (functionality).

The normal pituitary glands do not accumulate FDG and are not visualized by FDG-PET imaging of the human brain [27]. Among the intracranial structures of dogs, the pituitary gland has the second lowest FDG uptake; therefore, similar to the normal human pituitary gland, the normal canine pituitary gland cannot be visualized by FDG-PET imaging [28]. The detection of microtumors by commercial PET/CT is limited due to its low spatial resolution and partial-volume effect. To overcome these limitations and improve the accuracy of FDG uptake measurement in the pituitary gland, an HRRT-PET and 7 T MRI fusion imaging system was used for the evaluation of this case. 7 T MRI clearly visualized the pituitary lesion, whereas FDG uptake was not detected using PET. Therefore, FDG-PET did not enhance the diagnostic utility of 7 T MRI in the present case. However, negative findings could be observed in pituitary microtumors and FDG-PET was applied in only one case of pituitary microtumor in a dog. Additionally, the routine use of PET-MRI fusion imaging in PDH cases is difficult presently, because of no defined consensus on the interpretation of FDG uptake for the pituitary gland in veterinary medicine. Therefore, further investigation of this imaging technique is necessary in dogs with PDH, especially in CT or MRI negative cases.

## Abbreviations

ACTH: Adrenocorticotropic hormone
CT: Computed tomography
FDG: ^18^F-fluorodeoxyglucose
FOV: Field of view
HDDST: High-dose dexamethasone suppression test
HRRT: High-resolution research tomography
MRI: Magnetic resonance imaging
PDH: Pituitary-dependent hyperadrenocorticism
PET: Positron emission tomography
SUV: Standardized uptake value
WI: Weighted images
